# Effect of snack-food proximity on intake in general population samples with higher and lower cognitive resource

**DOI:** 10.1016/j.appet.2017.11.101

**Published:** 2018-02-01

**Authors:** Jennifer A. Hunter, Gareth J. Hollands, Dominique-Laurent Couturier, Theresa M. Marteau

**Affiliations:** Behaviour and Health Research Unit, Institute of Public Health, Forvie Site, University of Cambridge School of Clinical Medicine, Box 113 Cambridge Biomedical Campus, Cambridge, CB2 0SR, United Kingdom

**Keywords:** Proximity effect, Snack-food, Cognitive resource, Education level, Intervention

## Abstract

**Objective:**

Placing snack-food further away from people consistently decreases its consumption (“proximity effect”). However, given diet-related health inequalities, it is important to know whether interventions that alter food proximity have potential to change behaviour regardless of cognitive resource (capacity for self-control). This is often lower in those in lower socio-economic positions, who also tend to have less healthy diet-related behaviours. Study 1 aims to replicate the proximity effect in a general population sample and estimate whether trait-level cognitive resource moderates the effect. In a stronger test, Study 2 investigates whether the effect is similar regardless of manipulated state-level cognitive resource.

**Method:**

Participants were recruited into two laboratory studies (Study 1: *n* = 159; Study 2: *n* = 246). A bowl of an unhealthy snack was positioned near (20 cm) or far (70 cm) from the participant, as randomised. In Study 2, participants were further randomised to a cognitive load intervention. The pre-specified primary outcome was the proportion of participants taking any of the snack.

**Results:**

Significantly fewer participants took the snack when far compared with near in Study 2 (57.7% vs 70.7%, β = −1.63, *p* = 0.020), but not in Study 1 (53.8% vs 63.3%, X^2^ = 1.12, *p* = 0.289). Removing participants who moved the bowl (i.e. who did not adhere to protocol), increased the effect-sizes: Study 1: 39.3% vs 63.9%, X^2^ = 6.43, *p* = 0.011; Study 2: 56.0% vs 73.9%, β = -2.46, *p* = 0.003. Effects were not moderated by cognitive resource.

**Conclusions:**

These studies provide the most robust evidence to date that placing food further away reduces likelihood of consumption in general population samples, an effect unlikely to be moderated by cognitive resource. This indicates potential for interventions altering food proximity to contribute to addressing health inequalities, but requires testing in real-world settings.

**Trial registration:**

Both studies were registered with ISRCTN (Study 1 reference no.: ISRCTN46995850, Study 2 reference no.: ISRCTN14239872).

## 1 Introduction

Populations of lower, compared with higher, socio-economic position (SEP) consume more energy-dense foods (Monsivais & Drewnowski, 2009) and fewer fruits and vegetables (Stringhini et al., 2011), a suboptimal diet contributing to poor health at population level (Newton et al., 2015). Specifically concerning education level, being one indicator of SEP, those with lower, compared with higher, education levels consume less fruits and vegetables, more red and processed meats and more sugar (Maguire & Monsivais, 2015). These findings highlight the need for effective interventions to improve diet in these groups. Of concern is evidence that interventions that rely on providing information to change behaviour are more likely to benefit those of higher SEP, i.e. those with higher education, income and occupational levels (Beauchamp et al., 2014, Lorenc et al., 2013, McGill et al., 2015), exacerbating observed inequalities in health. Conversely, interventions that alter structural cues in the environment, thought to operate largely outside of awareness, have potential to reduce health inequalities (Hollands et al., 2016, Marteau et al., 2012).

One factor that may moderate outcomes of information-based interventions is cognitive resource, a term encompassing mental processes including intelligence and executive functions (EF), the latter of which is involved in planning and regulating thoughts and behaviour (Diamond, 2013). Indicators of lower SEP such as greater financial strain and lower maternal education level during early years of development and over the life course have a negative impact on trait-level cognitive resource (Raver et al., 2013, Singh-Manoux et al., 2005) and associated brain structures (Noble et al., 2015). Sustained poverty throughout young adulthood predicts poorer cognitive function in midlife (Al Hazzouri, Elfassy, Sidney, Jacobs, & Yaffe, 2017). SEP negatively impacts state-level cognitive resource, with people from lower income groups showing poorer impulse control (Mani, Mullainathan, Shafir, & Zhao, 2013) and greater vulnerability to unhealthy food advertising when under temporary cognitive load (Zimmerman & Shimoga, 2014). Lower cognitive resource, such as EF, is associated with overeating and higher BMI in young people (Groppe and Elsner, 2015, Reinert et al., 2013) and lower quality food choice in adulthood (Cohen et al., 2011, Hall, 2012). Despite this evidence, intervention studies rarely explore differential outcomes by SEP (McGill et al., 2015) or cognitive resource. Given the cognitive effort required to translate health information into sustained behaviour change, differences in cognitive resource by SEP could explain the evidence that information-based interventions may contribute to diet-related health inequalities. In contrast, if interventions that alter environmental cues do not rely on cognitive resource for their impact, they may be less likely to widen existing inequalities and may even reduce them if more effective in those with lower cognitive resource.

There are a variety of environmental cues that can be manipulated to shape diet-related behaviours (Hollands et al., 2013, Hollands et al., 2017a), such as the distance at which food is positioned. Increasing the distance between food and people decreases the likelihood that they select and consume it (for reviews see Bucher et al., 2016, Hollands et al., 2017b; see also Baskin et al., 2016, Kroese et al., 2015, Musher-Eizenman et al., 2010, Meyers and Stunkard, 1980; Levitz, 1976) and this has been observed across a range of foods including chocolate, desserts, savoury snacks and sliced fruits and vegetables. This “proximity effect” seems consistent regardless of craving (Maas, de Ridder, de Vet, & de Wit, 2012) and food preferences (Privitera & Zuraikat, 2014) and occurs even when increases of distance are relatively small e.g. 25.4 cm (Rozin et al., 2011) or 50 cm (Maas et al., 2012). More distant snacks, that require people to reach for them, are rated as more effortful to obtain compared to closer snacks (Maas et al., 2012). Since the least effortful course to obtain food is considered the most likely, placing unhealthy foods further away should reduce their intake without relying on explicit instruction or conscious deliberation by the actor (Marteau et al., 2012). This means that, in theory, such an intervention should be similarly effective at changing dietary behaviour in populations with lower as well as higher cognitive resource.

Current evidence for whether the proximity effect is moderated by cognitive resource is limited. First, sample populations are not representative of general populations, with most studies recruiting primarily university staff and students (Maas et al., 2012, Meiselman et al., 1994, Painter et al., 2002, Privitera and Creary, 2013, Privitera and Zuraikat, 2014, Rozin et al., 2011, Wansink et al., 2006). These populations have higher education levels, indicating higher SEP, and thus likely have higher levels of cognitive resource. Second, the quality of existing studies is compromised by small sample sizes and absence of power calculations (e.g. Maas et al., 2012, Painter et al., 2002, Privitera and Creary, 2013, Privitera and Zuraikat, 2014, Wansink et al., 2006) which limit the reproducibility of the effects found in many studies (Munafó et al., 2017, Button et al., 2013). Studies recruiting larger samples in general populations will provide more reliable and generaliseable estimates of the magnitude of the proximity effect (Bucher et al., 2016). Furthermore, to improve the reproducibility of existing studies and ensure quality-control and transparency of future research, studies should be pre-registered and study protocols and related information made available to other researchers (Munafó et al., 2017, Button et al., 2013).

To date, the hypothesis that altering environmental cues shapes eating behaviour in all recipients, irrespective of cognitive resource, remains largely untested (Hall & Marteau, 2014). As far as we are aware, no studies have investigated whether the proximity effect is moderated by cognitive resource. Such an investigation may determine whether the proximity effect has potential to improve diet in lower as well as higher SEP groups. Ascertaining whether any effect is evident regardless of cognitive resource could inform efforts to develop interventions that avoid increasing existing inequalities in dietary behaviour at population level.

The current studies build on existing literature: first, by estimating the magnitude of the proximity effect in larger general population samples, including those with lower education level (as an indicator of SEP), by replicating and extending an existing study conducted in a smaller university student sample (Maas et al., 2012), and second, by providing preliminary evidence for whether the proximity effect is moderated by cognitive resource. In line with previous research (Maas et al., 2012), the studies also assess effort as a possible underlying mechanism of the proximity effect.

## 2 Study 1

### 2.1 Methods

Further details of the methods used for Study 1 can be found in the published study protocol (Hunter, Hollands, Couturier, & Marteau, 2016).

#### 2.1.1 Hypotheses

1. A lower proportion of participants will take the snack food when it is placed far (70 cm) compared to when it is placed near (20 cm) to them.
2. The proximity effect will not be moderated by cognitive resource.

#### 2.1.2 Study design and setting

Participants were randomly allocated to one of two conditions using a between-subjects experimental design:

1. Snack bowl is placed near (20 cm)
2. Snack bowl is placed far (70 cm)

**Fig. 1:**
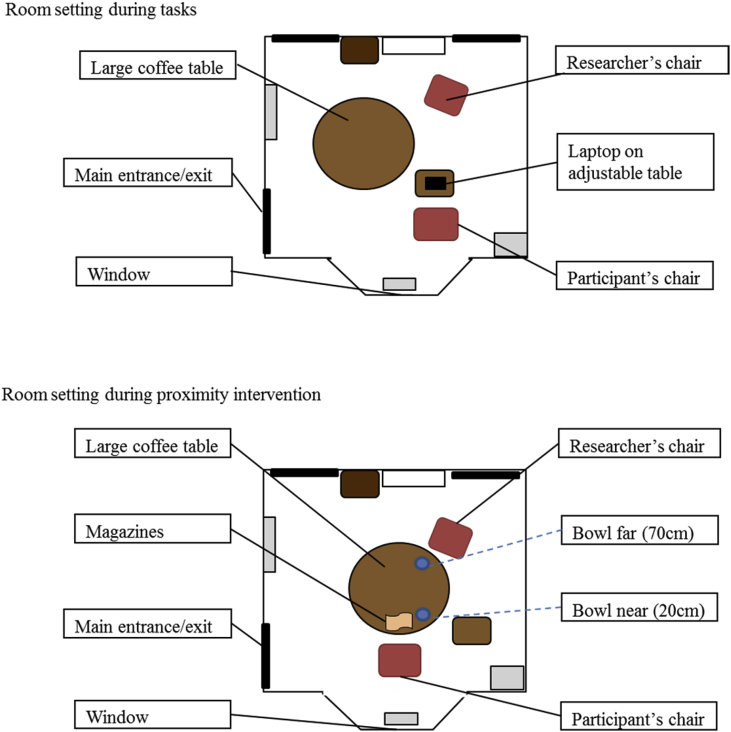
Map of the testing room.

Participants were tested individually in sessions running between 9am and 8pm in a multi-purpose room – see Fig. 1 for a map of the testing room.

#### 2.1.3 Participants

Members of the general public aged 18 and above were recruited from the Cambridgeshire (UK) area by a research agency using online, phone and on-street methods. The agency was instructed to recruit equal numbers of participants from higher and lower education levels. Those reporting relevant food allergies were excluded. Based on the aggregate results of the two studies conducted by Maas et al. (2012) giving probabilities of participants selecting any amount of the snacks of 0.76 in the near condition and 0.39 in the far condition, we calculated a required sample of 56 participants (28 in each study arm) to detect a main effect of distance on the proportion of participants consuming any amount of snacks with 80% power at the 0.05 level using logistic regression analysis. We aimed to recruit 156 participants to increase the study power to detect an effect on the primary and secondary outcomes.

#### 2.1.4 Food distance intervention

**Fig. 2:**
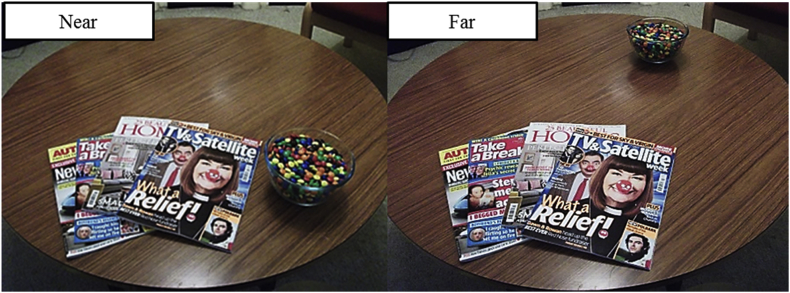
Snack presentation in each distance condition.

Participants were provided with 1000 g of chocolate M&Ms presented in a clear glass 1-L bowl. The bowl was placed either 20 cm (near) or 70 cm (far) from the seated participants’ right arm. See Fig. 2 for an image of the table layout in each distance condition.

#### 2.1.5 Outcomes and measures

*Primary outcome.* As pre-specified in the protocol (Hunter et al., 2016), the primary outcome consisted of the proportion (%) of participants who took any snacks, assessed by measuring the difference in bowl weight before and after the relaxation break.

*Secondary outcome.* The mean amount of snacks consumed (grams), was measured from the absolute difference in bowl weight from before to after the relaxation break.

*Cognitive resource.* This was assessed behaviourally using the Stroop colour-word interference task (Stroop, 1935), requiring participants to respond as quickly and as accurately as possible to a series of colour-words. The Stroop task was selected since it demonstrates adequate to good test-retest reliability (*r* = 0.68 to 0.91: Van Der Elst et al., 2008, Beglinger et al., 2005) and is sensitive to detecting variations in executive function associated with variations in brain structure (Cohen et al., 2011, Homack and Riccio, 2004), indicating that it assesses trait-level cognitive resource. This task requires participants to inhibit their responses to colour-words whose colour and meaning are incongruent. Participants’ interference score (in milliseconds (ms)) was used as the primary outcome of the Stroop task, which has been used widely as a measure since Stroop (1935). Interference score was calculated using the following equation: *mean incongruent latency – [mean congruent latency + mean control trial latency]/2* (Van der Elst, Van Boxtel, Van Breukelen, & Jolles, 2006). Mean reaction time (ms) for correct responses to incongruent stimuli (when colour words ink and meaning did not match) was used for exploratory analysis, since we found this sensitive to education level in both studies.

*Participant snack bowl manipulation.* Participants who moved the bowl undermined fidelity of the study protocol. Any bowl manipulation by the participant was recorded and considered in the analysis.

*Education level.* A dichotomous variable was defined by education level where participants obtaining up to 5 or more GCSEs/1 A-level were classified as lower education level and participants obtaining 2 or more A-levels and above were classified as higher education level.

*Additional outcomes.* Further details on outcomes (hunger, liking for chocolate, effort to obtain the snack and salience of the snack) can be found in the published study protocol (Hunter et al., 2016).

#### 2.1.6 Procedure

Ethics committee approval was sought and obtained from the University of Cambridge Psychology Research Ethics Committee (Pre.2015.008). Participants were recruited and screened for eligibility by the research agency using a questionnaire. Eligible participants were invited to take part in the experimental session and allocated an appointment by the agency. All participants gave informed consent prior to completion of the screening questionnaire, and again on entry to the experimental session. Participants were recruited into a study of “relaxation and personality”, creating a cover story in which the snack bowl could be presented unobtrusively. At the start of the session, participants provided baseline measures of cognitive resource and were then given their ten-minute “relaxation break” during which the snack bowl was placed on the table at the set distance pre-specified by the condition along with a selection of magazines. Before leaving the participant alone in the room, the researcher informed the participant that they were free to read the magazines and help themselves to the snacks. Following the break, the researcher returned to the room and the participant repeated the measures of cognitive resource to give the impression that the relaxation break served as an intervention of relaxation, giving credence to the cover story. Participants then completed the questionnaire requesting ratings of hunger, liking for chocolate and ratings of effort and salience of the snack foods used in the study. Finally, participants were verbally debriefed about the true nature of the study, given a debriefing sheet including researcher contact information and reimbursed.

#### 2.1.7 Analysis

For Hypothesis 1, an intention to treat analysis (including all participants as randomised) was conducted for the primary outcome (proportion of participants taking the snack) and the secondary outcome (total quantity of the snack consumed in participants who took the snack). To assess the primary outcome, 2-sample tests for equality of proportions and logistic regression analyses were conducted to investigate any between-group differences by distance condition on the proportion of participants who took any snacks. The amount of the snack consumed in grams followed a semi-continuous distribution with an excess of zeros, corresponding to participants who did not take any snack, and a skewed distribution for the strictly positive outcomes. T-tests and regression analyses on the log of the mean amount of snacks consumed were conducted, assuming that the amount of snack consumed in grams follows a zero-inflated log-normal distribution. Exploratory sensitivity analysis was conducted excluding participants consuming more than 3 standard deviations above the mean amount of the snack consumed to assess whether the secondary outcome was sensitive to outliers.

For Hypothesis 2, interaction terms for cognitive resource and proximity condition were included in regression analyses to investigate whether the proximity effect was moderated by cognitive resource. Participants taking longer than three standard deviations above the mean time taken (ms) or less than 200 ms to respond to colour-words were excluded from the analysis (*n* = 4) (based on Strauss, Allen, Jorgensen, & Cramer, 2005). Because a number of participants moved the bowl (i.e. did not adhere to the intended protocol), a sensitivity analysis was conducted for Hypothesis 1, excluding data from these participants. This analysis was exploratory for Study 1 as it had not been explicitly specified prior to any data analysis (although it was pre-specified for Study 2). Further to the analysis to test Hypothesis 2, to investigate any moderation by education level on the proximity effect, separate logistic (proportion of participants taking snacks) and log-normal (amount of snacks taken) regression analyses were conducted with interaction terms for distance condition and education level. Finally, Mann-Whitney-Wilcoxon tests were conducted to investigate any effects of the distance intervention on perceived effort and salience of the snack food. All analyses were conducted using SPSS version 22 and R version 3.2.2.

### 2.2 Results

#### 2.2.1 Participant characteristics

A total of 159 participants were tested (*n* = 65 lower education level and *n* = 94 higher education level), see Table 1 for sample population characteristics. Participants with lower education level demonstrated slower reaction time in the Stroop task compared to those with higher education level, *U* = 2,299, *p* = 0.033. A total of 66 (41.5%) participants did not take any of the snack.

**Table 1:** Demographic and baseline characteristics of the study sample.

Characteristics	Condition		All participants (*N* = 159)
	Near (*n* = 79)	Far (*n* = 80)	
Age (*M* (*SD*))	38.8 (15.6)	38.0 (14.8)	38.4 (15.2)
Gender (%(*n*))			
Male	35.4 (28)	37.5 (30)	36.5 (58)
Female	64.6 (51)	62.5 (50)	63.5 (101)
BMI (*M* (*SD*))	24.8 (4.8)	24.7 (3.8)	24.8 (4.3)
Education (%(*n*))			
<4 GCSEs	17.7 (14)	30.0 (24)	23.9 (38)
>5 GCSEs/1 A-level	15.2 (12)	18.8 (15)	17.0 (27)
>2 A-levels/Degree	48.1 (38)	35.0 (28)	41.5 (66)
Postgraduate degree	19.0 (15)	16.3 (13)	17.6 (28)
Ethnicity (%(*n*))			
White	79.7 (63)	90.0 (72)	84.9 (135)
Mixed	2.5 (2)	6.3 (5)	4.4 (7)
Asian	12.7 (10)	2.5 (2)	7.6 (12)
Black	1.2 (1)	0.0 (0)	0.6 (1)
Other/rather not say	3.8 (3)	1.3 (1)	2.5 (4)
Stroop (*M* (*SD*))			
Baseline reaction time (ms)	1844.6 (797.9)	1831.1 (898.5)	1837.8 (847.9)
Baseline interference (ms)	308.6 (310.8)	268.9 (291.2)	288.6 (300.8)
Liking for chocolate (*M* (*SD*))	32.6 (27.2)	37.3 (30.7)	35.0 (29.0)
Hunger (*M* (*SD*))	2.2 (1.3)	2.9 (1.7)	2.5 (1.5)

#### 2.2.2 Randomisation checks

We checked if the distribution of eight potential predictors was equal between distance conditions using X^2^ or Fisher tests for categorical predictors and Wilcoxon tests for continuous predictors. Hunger level differed with statistical significance between distance conditions, while education level and ethnicity differed but this was statistically non-significant. These variables were therefore included as covariates in sensitivity analyses. No other differences in participant characteristics between distance conditions were found. Note that no significant difference between groups would have been detected when using a Bonferroni multiplicity correction.

#### 2.2.3 Effect of distance on snack-food intake

A lower proportion of participants took any of the snack in the far (53.8%) compared to the near condition (63.3%). The direction of the effect was consistent with Hypothesis 1 although the overall effect was small and statistically non-significant (*d* = 0.22) – see Table 2. For the secondary outcome, participants took 27.3 g (*SD* = 26.0) of the snack when the bowl was placed further away compared to 26.3 g (*SD* = 29.9) when nearer, *t* (91) = −0.61, *p* = 0.546, *d* = −0.13, an effect that was not sensitive to inclusion of control variables or exclusion of outliers.

**Table 2:** Proportion (%(*n*)) of participants taking snacks in each distance condition.

	Condition				Effect without control variables^a^	Effect with control variables^a^
	Near (*n* = 79)		Far (*n* = 80)			
All participants	63.3	(50)	53.8	(43)	X^2^ = 1.12, *p* = 0.289	β = −0.39, *p* = 0.256,
	Near (*n* = 61)		Far (*n* = 61)			
Excl. bowl movers	63.9	(39)	39.3	(24)	X^2^ = 6.43, *p* = 0.011	β = −0.91, *p* = 0.021,

a Without control variables, the X^2^-statistic of a 2-sample test for equality of proportions with continuity correction and corresponding p-value is reported. When controlling for the variables education level, hunger and ethnicity, the estimated logistic regression coefficient for the distance effect (β) and its corresponding p-value are reported.

#### 2.2.4 Cognitive resource and the proximity effect

No interaction was found between Stroop interference and distance condition on the proportion of participants taking snacks, β = 0.0004, *p* = 0.756, or for the log-amount of snacks taken, β = 0.0001, *p* = 0.837.

#### 2.2.5 Additional analysis

Participants who moved the bowl were more likely to take the snack than those who did not, 81.1% vs. 51.6%, X^2^ = 8.96, *p* = 0.003. Following exclusion of participants who moved the bowl (*n* = 37), a logistic regression analysis was conducted (*n* = 122), showing a statistically significant effect of distance on the proportion of participants who took M&Ms - see Table 2: a lower proportion of participants took any of the snack in the far (39.3%) compared to the near condition (63.9%), a larger statistically significant proximity effect (*d* = 0.56). Sensitivity analysis with regression models with and without the control variables did not affect these results - see Table 2. Participants who moved the bowl consumed more than those who did not, 40.5 g (SD = 32.1) vs. 20.2 (SD = 23.4), *t* (91) = −3.68, *p* < 0.001. When participants who moved the bowl were excluded from the analysis, the effect of proximity on the amount of the snack consumed remained statistically non-significant with 17.2 g (*SD* = 15.1) of the snack consumed when further away compared to 22.1 g (*SD* = 27.2) when closer, *t* (61) = 0.40, *p* = 0.693, *d* = 0.10, an effect not sensitive to inclusion of control variables or the exclusion of outliers.

There was no interaction between education level and distance for the proportion of participants taking snacks, β = 0.18, *p* = 0.790, or the amount of snacks taken, β = 0.09, *p* = 0.655. Participants in the far condition rated taking snacks as more effortful, *M* = 2.93 (*SD* = 0.98), than those in the near condition, *M* = 1.79 (*SD* = 0.77), *U* = 5,131, *p* < 0.001. Perceived salience did not differ between the near, *M* = 3.13 (*SD* = 1.23) and far conditions, *M* = 3.13 (*SD* = 1.24), *U* = 3,168, (*p* = 0.978).

### 2.3 Study 1 discussion

Study 1 did not replicate the proximity effect, finding a small statistically non-significant effect. However, when participants who moved the bowl (i.e. who did not adhere to the intended protocol) were excluded from the analysis, significantly fewer participants took any of the snack when it was further away, consistent with Hypothesis 1. No proximity effect was found on the amount of the snack consumed. It is possible that a general population sample is affected differently by food distance compared to university samples in previous studies. Consistent with these studies, participants rated distant snacks as more effortful to obtain, indicating effort may be an underlying mechanism of the proximity effect.

Study 1 was limited in four ways: first, the proximity effect was attenuated when participants moved the bowl, suggesting that a more appropriate test of the effect of proximity requires fixing the snack bowl's position. Second, the smaller sample sizes per condition following removal of participants who consumed nothing may have reduced the statistical power to assess the amount of the snack consumed. Third, testing Hypothesis 2 was exploratory due to inadequate statistical power to investigate any interaction between cognitive resource and distance condition, an investigation which required an unrealistic sample size of 508, assuming a moderate effect size. Furthermore, the way Hypothesis 2 is stated assumes that the null hypothesis is true. If rejecting it would prove it wrong, not rejecting it would not prove it right. Therefore, a suitably powered study in respect of a re-expression of Hypothesis 2 is required. Fourth, any moderation by cognitive resource, defined by Stroop outcomes, could have been due to other cognitive resources associated with this trait, such as intelligence (Arffa, 2007) and education level (Moffitt et al., 2011).

## 3 Study 2

### 3.1 Methods

Study 2 aimed to address the limitations of Study 1 with the following adjustments. First, the bowl was placed on a non-slip mat to increase the effort participants required to move the bowl, thus reducing the chance it is moved. Second, Study 2 recruited a larger sample and used a within-subjects design comparing higher and lower cognitive resource, increasing statistical power to investigate cognitive resource and the amount of the snack taken. Finally, Study 2 aimed to manipulate state-level cognitive resource to provide a stronger test of whether the proximity effect is moderated by cognitive resource.

Further details on the methods are provided in the Study 2 protocol, which is available online [10.6084/m9.figshare.4960193].

#### 3.1.1 Hypotheses

1. A lower proportion of participants will take the snack food when it is placed far (70 cm) compared to when it is placed near (20 cm) to them
2. The proportion of participants taking snacks will be equivalent at each given snack-bowl distance regardless of participants' cognitive load.

#### 3.1.2 Study design and setting

Participants were tested in the same room as used in Study 1. The experimental sessions lasted one hour, taking place between 11am and 8pm. The study used a mixed design in which participants were randomly allocated to one of four conditions to receive a snack placed at a fixed distance (near or far) throughout the session (between-subjects) and were under cognitive load (load or no load) either in the first or second half of the session (within-subjects):

1. Snack near (20 cm), cognitive load in the second half of the session
2. Snack near (20 cm), cognitive load in the first half of the session
3. Snack far (70 cm), cognitive load in the second half of the session
4. Snack far (70 cm), cognitive load in the first half of the session

#### 3.1.3 Participants

Participants aged 18 years and over were recruited from the same general population as in Study 1 using the same agency, methods and criteria. The agency ensured that people who had participated in Study 1 were not recruited into Study 2. Based on equivalence testing using the Two One-Sided Test (TOST) approach (Tango, 1998), the zone of indifference (*i.e.* the zone in which two parameters are considered equivalent) is defined as ±0.191 (for the near condition) and ±0.189 (for the far condition). This is taken from the estimated proportion of participants taking snacks per distance condition from those observed in Study 1 (near = 0.639 and far = 0.393). This zone was defined only for the sample size calculation and assumes the worst case scenario *i.e.* if a smaller correlation of 0.5 were detected. Assuming a power of 80% and a significance level of 0.05, we estimated a required sample size of 230 participants (giving a total of 460 observations, 115 in each study arm) to detect equivalence of proportion taking snacks per cognitive manipulation condition at each given distance from the snack bowl.

#### 3.1.4 Food distance intervention

**Fig. 3:**
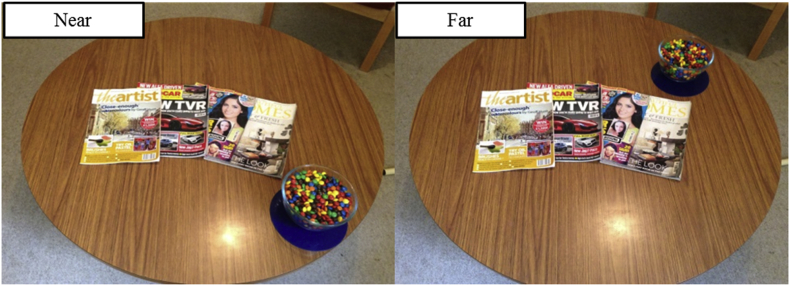
Snack presentation in each distance condition.

The intervention was the same as used in Study 1. See Fig. 3 for the layout of the table in each condition.

#### 3.1.5 Cognitive load manipulation

Participants were instructed to memorise 7 digits, and told they had to recall these in the correct order at some point during the study session. This task has been found to impact dietary behaviour resulting from an apparent reduction in cognitive resource (Shiv and Fedorikhin, 1999, Van Dillen et al., 2013, Zimmerman and Shimoga, 2014). The digits were displayed centrally on a laptop for ten seconds.

#### 3.1.6 Measures

All measures are identical to those used in Study 1, apart from the addition of the 15-item version of the Barratt Impulsivity Scale (BIS15: Spinella, 2007). Each item was rated on a 4-point rating scale anchored by 1 = rarely/never to 4 = almost always. A higher total score indicated greater impulsivity.

#### 3.1.7 Procedure

Ethics committee approval was sought and obtained from the University of Cambridge Psychology Research Ethics Committee (Pre. 2016.028). The procedures used for informed consent, recruitment and the experimental session were identical to Study 1 with the following adjustments. First, the session was repeated twice to satisfy the within-subjects nature of the cognitive load manipulation, which was given either in the first or second half of the session depending on randomisation. Second, participants completed the Stroop task at the start and end of each half of the session to assess the impact of cognitive load on cognitive resource. Finally, participants completed hunger and food ratings at the end of the second half of the session. At the end of the session, participants were verbally debriefed about the true nature of the study, and given a debriefing sheet including researcher contact information, and reimbursed.

#### 3.1.8 Analysis

To test Hypothesis 1, General linear mixed models (GLMM) were conducted using R Studio version 3.3.1. (lme4 package version 1.0.+) to investigate any differences by distance condition on the proportion of participants consuming any of the snack and the amount consumed. Exploratory sensitivity analysis was conducted excluding participants consuming more than 3 standard deviations above the mean amount of the snack consumed to assess whether the secondary outcome was sensitive to outliers. To test Hypothesis 2, all participants across both halves of the session were analysed together (*n* = 246). Equivalence testing was conducted using a Two One-Sided Test (TOST) approach to assess whether the proportion of participants taking any amount of the snack was equivalent at each given bowl-distance, regardless of whether participants were under cognitive load. Using the 95% score confidence intervals (Tango, 1998) calculated for each distance condition (near = −0.069 to 0.085, far = −0.042 to 0.109), the zone of indifference was defined as ±0.127, based on a tetrachoric correlation of 0.90 and a sample size of 246. For participants who did not move the bowl, the zone of indifference was redefined as ±0.133, based on a lower sample size of 227. In addition to analysis for Hypothesis 1, as pre-specified in the protocol, analysis was conducted for all participants and additionally only for those participants who did not move the bowl. Separate regression analyses were carried out to investigate whether distance condition and education level showed an interaction affecting the proportion of participants taking any of the snack (logistic regression) and the amount taken per consumer (log-normal regression). Finally, Mann-Whitney-Wilcoxon tests were used to investigate any effects of distance on perceived effort and salience of the snack food.

Since previous studies using digit-memorisation have typically only assumed, rather than actually tested, an effect on cognitive resource (Shiv and Fedorikhin, 1999, Van Dillen et al., 2013, Zimmerman and Shimoga, 2014), the effect of this manipulation was assessed directly by comparing Stroop performance before and after receiving cognitive load as a manipulation check. Participants taking longer than three standard deviations above the mean time taken (ms) or less than 200 ms to respond to colour-words were excluded from the analysis (*n* = 2 at baseline, *n* = 6 post-manipulation).

### 3.2 Results

#### 3.2.1 Participant characteristics

A total of 246 participants were tested (*n* = 107 lower education level and *n* = 139 higher education level), see Table 3 for sample population characteristics. Participants with a lower education level demonstrated slower Stroop reaction time compared with those with a higher education level, *U* = 4,709, *p* < 0.001. A total of 106 (43.1%) participants did not take any of the snack.

**Table 3:** Characteristics of participants by distance condition.

Variables	Condition		
	Near (*n* = 123)	Far (*n* = 123)	Total (*N* = 246)
Gender (%(*n*))			
Male	41.5 (51)	44.7 (55)	43.1 (106)
Female	58.5 (72)	54.5 (67)	56.5 (139)
Other	0.0 (0)	0.8 (1)	0.4 (1)
BMI (*M* (*SD*))	25.6 (5.5)	25.8 (5.6)	25.7 (5.5)
Age (*M* (*SD*))	35.7 (12.7)	36.8 (13.2)	36.2 (13.0)
Education (%(*n*))			
<4 GCSEs	17.1 (21)	18.7 (23)	17.9 (44)
>5 GCSEs/1 A-level	25.2 (31)	26.0 (32)	25.6 (63)
>2 A-levels/Degree	31.7 (39)	32.5 (40)	32.1 (79)
Post-graduate degree	26.0 (32)	22.8 (28)	24.4 (60)
Ethnicity (%(*n*))			
White	87.0 (107)	88.6 (109)	87.8 (216)
Mixed	4.9 (6)	1.6 (2)	3.3 (8)
Asian	4.9 (6)	4.1 (5)	4.5 (11)
Black	2.4 (3)	4.1 (5)	3.3 (8)
Other/rather not say	0.8 (1)	1.6 (2)	1.2 (3)
Stroop (*M* (*SD*))			
Baseline reaction time (ms)	1659.8 (743.8)	1607.9 (651.0)	1634.1 (698.3)
Baseline Interference (ms)	322.7 (326.3)	269.8 (233.7)	296.4 (284.7)
Liking for chocolate (*M* (*SD*))	34.7 (29.0)	36.8 (30.5)	35.8 (29.7)
Hunger (*M* (*SD*))	3.0 (1.6)	2.9 (1.7)	2.9 (1.7)
Impulsivity (*M* (*SD*))	32.1 (6.3)	32.3 (6.6)	32.2 (6.5)

#### 3.2.2 Randomisation checks

Randomisation by distance condition was successful with no differences observed in participant characteristics by condition. Age was included as a covariate in all analyses since this was associated with baseline Stroop reaction time, *r* (244) = 0.454, *p* < 0.001, and interference, *r* (245) = 0.270, *p* < 0.001. Hunger was included as a covariate since hunger ratings differed by cognitive load condition, though this was statistically non-significant.

#### 3.2.3 Manipulation checks

The analysis of the reaction times by means of linear mixed models including load and session as fixed effects and participants as random intercepts showed that reaction times are influenced by both load, which increases reaction times by 141.2ms on average (*z* = 2.83, *p* = 0.005), and practice (on average, participants reacted 99.3 ms faster at session 2 compared to session 1 when controlling for load (*z* = −1.99, *p* = 0.047)) while the interaction between load and practice was not significant (β = −158.4, *z* = −1.69, *p* = 0.091).

#### 3.2.4 Effect of distance on snack-food intake

Consistent with Hypothesis 1, there was a statistically significant effect of distance on whether participants took any of the snack (*d* = 0.32), with a lower proportion of participants taking any of the snack in the far condition (57.7%) compared to the near condition (70.7%). There was no difference in the proportion of snack takers between participants who moved the bowl and those who did not. The proximity effect was stronger when participants who moved the bowl (*n* = 20) were excluded from the analysis (*d* = 0.44) - see Table 4.

**Table 4:** Proportion (%(*n*)) of participants taking snacks in each distance condition.

	Condition				Effect without control variables	Effect with control variables
	Near (*n* = 123)		Far (*n* = 123)			
All participants	70.7	(87)	57.7	(71)	β = −1.63, *p* = 0.020	β = −1.62, *p* = 0.022
	Near (*n* = 111)		Far (*n* = 116)			
Excl. bowl movers	73.9	(82)	56.0	(65)	β = −2.46, *p* = 0.003	β = −2.59, *p* = 0.005

*Note:* The control variables included in the model were age and hunger. Note that in both regression models, cognitive load was included as a control variable. Those excluded were any participants who moved the bowl at least once over the two observations.

The amount of the snack participants took did not differ between the distance conditions: participants took on average 30.5 g (*SD* = 31.2) of the snack when the bowl was placed further away compared to 27.5 g (*SD* = 19.3) when nearer (β = −0.22, *p* = 0.615, *d* = −0.12), an effect not sensitive to control variables and with some sensitivity to exclusion of outliers (26.0 g (*SD* = 21.0) vs. 27.5 g (*SD* = 19.3), β = −0.61, *p* = 0.113, *d* = 0.08). This did not change when participants who moved the bowl were excluded from the analysis, 29.2 g (*SD* = 32.4) vs. 27.1 g (*SD* = 19.1), β = −0.32, *p* = 0.481, *d* = −0.08, an effect not sensitive to control variables but sensitive to the exclusion of *n* = 4 outliers, 24.1 g (*SD* = 21.0) vs.27.7 g (*SD* = 19.5), β = −0.78, *p* = 0.049, *d* = 0.18. Participants who moved the bowl consumed more than those who did not, 39.8 g (*SD* = 17.2) vs. 28.0 g (*SD* = 25.5), *t* (115) = −1.85, *p* = 0.067, though this effect was statistically non-significant.

#### 3.2.5 Cognitive load and distance condition

Consistent with Hypothesis 2, the proportion of participants who took any of the snack was statistically equivalent (95% CI was within ± δ = 0.127) when comparing across cognitive load conditions in both the near condition: (62.60% vs. 61.79%, 95% CI: −0.069; +0.085) and the far condition (51.21% vs. 47.97%, 95% CI: −0.041; +0.109). The proportion of snack takers was also equivalent following removal of participants who moved the bowl.

#### 3.2.6 Additional analysis

*Education level.* No interaction was found between distance condition and education level for either the proportion of participants who took any of the snack β = −0.38, *p* = 0.488, or the total amount of the snack taken, β = 0.04, *p* = 0.775.

*Perceived effort and salience of the snack food.* Participants in the far condition rated the snack as more effortful to obtain, *M* = 2.75 (*SD* = 1.00) compared to the nearer snack, *M* = 1.77 (*SD* = 0.79), *U* = 11,703, *p* < 0.001. A small statistically non-significant difference in salience was found between near (*M* = 5.73, *SD* = 1.93) and far conditions (*M* = 5.29, *SD* = 1.96, *U* = 6,563, *p* = 0.072).

### 3.3 Study 2 discussion

Participants were as likely to take snacks regardless of whether they were under cognitive load or not, consistent with the hypothesis that reducing the chance of food consumption by increasing food distance does not rely on cognitive resource to achieve this effect. However, Study 2 did not find a proximity effect on the amount of the snack taken.

Although the mixed design of Study 2 increased statistical power, its complexity introduced limitations. First, there may have been carryover effects of the cognitive load manipulation from the first to the second half of the session. This is supported by some participants under cognitive load in the first half reporting that they memorised the digits through the second half, despite already recalling the digits. Second, participants in Study 2 completed the Stroop task four times. Analysis described in the section “Manipulation checks”, showed that the repetition of Stroop tasks induced practice effects (which have been observed in the literature, see Strauss et al., 2005, Beglinger et al., 2005, Dikmen et al., 1999) that interfered with assessing the impact of cognitive load, which appeared as a weaker effect in the second half of the session. Despite this, the cognitive load had the expected depletory effect on cognitive resource. Third, the test of equivalence may have been less accurate when including participants for whom the cognitive load was less effective, possibly reducing the chance of detecting any effect of moderation by cognitive load emerging from the first half of the session. In sum, the study's power to assess whether the proximity effect operates regardless of cognitive resource may have been lower than expected.

Further research may benefit from a between-subjects design where participants complete the Stroop task fewer times. By eliminating carryover effects and reducing practice effects, the cognitive load manipulation should have the desired impact on resource in any future study, although a large sample will be required.

## 4 General discussion

To our knowledge, the current studies are the first to replicate the proximity effect in controlled experimental conditions in appropriately powered studies with general population samples (including those with lower as well as higher education level). The effect on consumption observed in these studies is therefore likely to be the most reliable to date. Furthermore, to our knowledge, these studies were the first to examine whether the impact of altering distance at which food is placed on consumption relies on cognitive resource, providing the first evidence that the proximity effect may not rely on conscious engagement to affect behaviour. Few participants reported awareness of any manipulation of the snack, supporting the non-conscious nature of the effect (Hollands et al., 2016). Both studies were novel in that they considered education level, an indicator of SEP, in the context of the proximity effect, indicating that the effect may operate similarly regardless of education level, though this requires further investigation.

Inconsistent with Maas et al. (2012), there was no proximity effect on the amount of the snack consumed, except in participants who did not move the bowl in Study 2 (when outliers were excluded). This may be due to limited statistical power of the study to detect an effect following removal of participants who did not consume, thus reducing the sample size. It is also possible that the proximity effect operates as a smaller effect in general population samples compared to student samples. A further possibility is that altering distance may not effectively reduce how much people consume once they have taken at least some of a product. A recent study has found that cognitive resource did not predict initiation of snack taking but did predict the amount consumed (Powell, McMinn, & Allan, 2017). Although no moderation by cognitive resource on the amount consumed was found in the current studies, there was not adequate statistical power to assess this. Further investigation is required into such possible moderation effects. Additionally, Study 1 identified the negative effect of participants moving the bowl on the fidelity of the distance intervention (although this analysis was exploratory in Study 1). This was mitigated in Study 2 by increasing the effort needed to move the bowl. Since it was uncertain whether moving the bowl occurred before or after the choice to take the snack, the subsequent bowl position may have impacted intake rather than just the initial bowl position, warranting the additional analyses excluding those who moved the bowl. Our findings suggest that the proximity effect is stronger when the food is at a fixed position, but weakened when participants are able to move the food. Future studies investigating the proximity effect should consider similar sensitivity analysis to determine whether participants altering the position of the product impacts the effect.

The current studies had several limitations. First, only education level was assessed, this being only one indicator of SEP, limiting the breadth of conclusions that can be drawn. In addition, the sample was not representative of the general population in either ethnicity or education level (with a large proportion of participants having postgraduate degrees). Second, only the Stroop task was used as an objective measure of cognitive resource, and may have captured state-level rather than trait-level cognitive resource. Future studies could consider administering multiple measures to attempt to assess trait-level cognitive resource. Third, since the session times inevitably varied, this likely led to variations in participants' hunger levels. Future studies should ask participants how hungry they felt at the start and end of the session in order to control for this. Fourth, participants’ usual dietary intake was not assessed, limiting our understanding of how this may have influenced their consumption. Fifth, the studies were conducted in artificial laboratory settings, testing participants in a novel context and providing a single unhealthy snack. This provides limited generalisability to real-world settings and further work is needed to inform development of useable interventions. For example, future laboratory studies could provide multiple foods differing in healthiness, a scenario more typical of real-world environments. Importantly, more field studies are needed within complex, uncontrolled environments, and future research should investigate whether impact of the proximity effect on intake is sustained in the longer-term (Bucher et al., 2016).

Given that reduced cognitive resource is linked to unhealthy diet, these results imply that an intervention altering food distance, which may operate non-consciously to affect behaviour, could inform efforts to tackle diet-related health inequalities at population level. The current studies indicate that placing unhealthy food an additional 50 cm further away increases effort required to obtain the food and has the potential to reduce chances of consumption. This effect could be capitalized on in designing real-world environments such as cafeterias or supermarkets, where products can be re-positioned to alter their degree of convenience for potential consumers e.g. moving less healthy foods from front to back rows of cafeteria buffet arrangements (Meyers & Stunkard, 1980), or away from till-points in shops (Kroese et al., 2015). While the significant potential of such interventions to impact on health-related behaviour at population-level is recognised, this remains relatively untested, with a cumulative evidence base that has been slow to develop (Hollands, Bignardi, et al., 2017).

## 5 Conclusions

The current studies provide the most robust evidence to date that placing food further away reduces likelihood of consumption in general populations and that this effect is unlikely to be moderated by cognitive resource. This indicates potential for interventions altering food proximity to contribute to addressing health inequalities, but requires further testing in real-world settings.

## Author contributions

TMM and GJH conceived the study and provided revisions to the manuscript. JAH planned and implemented the studies and drafted the manuscript under the supervision of TMM and GJH. DLC conducted the sample size calculations and contributed to the drafting of sample size calculation, planned analysis and results sections of the manuscript.

## Funding

This research was funded by the Medical Research Council (MRC) and Sackler Prize [MR/K50127X/1], awarded to JAH, The Department of Health Policy Research Program (Policy Research Unit in Behaviour and Health [PR-UN-0409-10109]), and National Institutes of Health [NF-SI-0513-10101] awarded to TMM. The funders had no role in the design of the studies, data collection and analysis, decision to publish, or preparation of the manuscript.

## Declaration of interest

The Authors declare they have no conflicts of interest.

## Compliance with ethical standards

All procedures performed in the studies were in accordance with the ethical standards of the institutional research committee and with the 1964 Helsinki declaration and its later amendments. Informed consent was obtained from all individual participants included in the studies.

## Appendix A Supplementary data

Data profile Data profile
